# Bridging gaps in healthcare: child health services and specialist care collaboration for young children with autism and coexisting conditions

**DOI:** 10.3389/fped.2025.1501650

**Published:** 2025-02-06

**Authors:** Gudrun Nygren, Christopher Gillberg, Emilia Carlsson

**Affiliations:** ^1^ Gillberg Neuropsychiatry Center, Sahlgrenska Academy, University of Gothenburg, Gothenburg, Sweden; ^2^ Child and Adolescent Specialist Centre, Angered Hospital, SV Hospital Group, Gothenburg, Sweden; ^3^ Institute of Neuroscience and Physiology, Sahlgrenska Academy, University of Gothenburg, Gothenburg, Sweden

**Keywords:** coordinated healthcare, collaboration, increased knowledge, training of medical staff, regulatory problems, autism, immigrant families

## Abstract

**Aim:**

This study aimed to evaluate a clinical project aiming to address gaps in healthcare for young children in an immigrant, low-resource district from early identification of regulatory problems, autism, and other neurodevelopmental symptoms by child health services to assessment and interventions in specialist care.

**Methods:**

A mixed-model design was employed, consisting of a description of the clinical project and data from healthcare statistics to evaluating the care chain. Qualitative in-depth interviews were conducted to capture the perspectives of participating child health nurses. Data were analyzed using content analysis.

**Results:**

The mean age for referral from primary to specialist care for suspected autism decreased from 38 to 27 months at (*n* *=* 59). A total of 55 children were diagnosed with autism. The mean age at autism diagnosis decreased from 44 to 31 months. Waiting times from referral to intervention were shortened. Interventions were already initiated in primary care at the time of referral. Qualitative analyses of nurse experiences revealed three main categories: (1) new and increased knowledge, (2) great importance for every child and family, and (3) an efficient method with fewer gaps, which holds further potential for development.

**Conclusion:**

Professionals’ increased knowledge of early symptoms in children, combined with novel healthcare methods for close collaboration, made it possible to bridge the gaps and provide young children and their families with early assessments and essential early interventions. The study results point to opportunities for integrated healthcare and collaboration with families and preschools.

## 1 Introduction

Healthcare faces significant challenges in meeting the diverse needs of young children and their families. In Sweden, 95%–99% (1) of children are regularly followed up through Child Health Services (CHS), which monitors the health and development milestones of children. However, there still remain delays in identifying early signs of autism and other neurodevelopmental disorders (NDDs). When autism and/or other NDDs are suspected, a referral is usually sent from primary care for further investigation at specialist units. A major general problem in many parts of our country and globally is long waiting times for evaluations, which can delay essential interventions for several years (2, 3). A recent study examining care pathways for children and adolescents with autism across three European countries reported significant shortcomings in healthcare, including long delays from initial concerns to screening and confirmed diagnosis, long waiting times for interventions, and no access to interventions (4). In addition to gaps in the care chain, there is often a lack of coordination with other parts of care regarding coexisting conditions, e.g., feeding difficulties and medical conditions, in young children with autism.

Early difficulties in crying, sleeping, and feeding, often referred to as regulatory problems (RPs), are common concerns in child health, affecting approximately 20% of infants (5).While these issues are only temporary for most children (5, 6), some children experience persistent RPs, which can cause great concern for the family and lead to frequent healthcare interactions. Several studies have drawn attention to persisting RPs beyond 3–4 months (7, 8) and the increased risk for continued developmental difficulties during childhood. These issues commonly occur in combination, e.g., sleeping and feeding problems. A Danish population study found persistent combined RPs in 8.6% of the children aged 8–11 months (7). A study from our group (8) found RPs in 50% of children with autism and, specifically, persisting RPs regarding feeding and eating in children who were later diagnosed with autism and continued eating difficulties. Given the increased risk for developmental problems, there is a need for early interventions and comprehensive follow-up for children with persisting RPs. A recent review by Asmussen et al. (9) pointed out the need for more research on the longitudinal relationship between combined RPs and mental health problems in children and the importance of investigating preventive interventions targeting persisting RPs.

Research emphasizes the importance of early identification of autism and early interventions (10). The core symptoms of autism, such as deficits in social communication and repetitive behaviours, can be observed as early as the first year of life, with most children displaying these symptoms between 12 and 24 months (11). Other early symptoms include sensory and motor deviations and regulatory problems (11, 12). Increased knowledge among professionals and screening programs in many countries have led to progress in the early identification of autism (10, 13, 14). Autism can be reliably diagnosed as early as 18 months of age (14, 15), and several studies have reported early parental concern around 18 months in children who are later diagnosed with autism (16, 17). However, there is often a delay of several years in diagnosis, and the median age of autism diagnosis is reported to be over the age of 4 years (2, 18).

The first years of life are considered a critical period, offering the best opportunities for interventions by significant neural plasticity (19). For children under 3 years of age, there is growing evidence for interventions covered under the umbrella term Naturalistic Developmental Behavioural Interventions (NDBI) (20, 21). These interventions integrate behavioural and developmental strategies within the child's naturalistic environment during daily activities. The most studied NDBIs is the Early Start Denver Model (ESDM) developed for children aged 12–48 months (22, 23, 24, 25). The ESDM is a comprehensive intervention focusing on the child's social-emotional, cognitive, and language development and broadly covering the different developmental areas. The individual child's motivation and interest are essential, and the learning context for the individualized interventions is embedded in child's daily activities in which adults create opportunities for learning and interaction through play activities and daily routines. Parental engagement is emphasized, and parents are trained to implement ESDM into their daily interactions with their children (26, 27).

Approximately 1% of children are diagnosed with autism around the world, but prevalence estimates vary significantly within and across sociodemographic groups (28). Autism is highly correlated to other NDDs, e.g., attention disorders, language disorders, motor disabilities, and intellectual disabilities (29, 30). To capture the different early manifestations of NDDs, affecting motor, social, and communication development, feeding, sleeping, and behavioural regulation, Gillberg coined the term ESSENCE (Early Symptomatic Syndromes Eliciting Neurodevelopmental Clinical Examinations). The ESSENCE concept underscores the need to follow-up and assess broadly when early concerning symptoms or disorders are identified. In the last few decades, more attention has also been paid to coexisting medical conditions in autism, which may influence the child's behaviour and need targeted treatments (14, 31, 32).

The biological basis of autism is heterogeneous and complex (33). Twin and family studies have demonstrated that genetic factors play a major role (34, 35). Various environmental factors, including pre- and perinatal factors and epigenetic mechanisms, have been proposed to interact and contribute to the manifestation of atypical neurodevelopmental patterns in autism (36, 37). However, the role of environmental factors is still not fully understood. In the last decade, several studies have indicated an association between maternal migration and the risk of autism (38, 39). In a previous study by our group, we reported a high prevalence of autism, 3.7%, among preschool children in an immigrant population (40). The high prevalence and the many needs of children and families in immigrant populations served as the basis for this study.

Barriers in healthcare service systems, including long waiting lists and separate disconnected units, pose significant challenges for young children and their families, hindering their access to timely assessments and early essential interventions (3, 4, 41). The situation is even more difficult for immigrant families in low-resource settings (28, 42). These healthcare barriers served as the catalyst for the clinical project examined in this study.

The aim of the study was to evaluate the novel methods within the clinical project to address gaps in healthcare. An additional aim was to explore the experiences of new methods and structures in CHS from the perspectives of child health nurses.

## 2 Methods

The study follows a mixed-model design, consisting of data from medical statistic charts and qualitative interviews to capture the perspectives of the included nurses.

### 2.1 Study context and participants

#### 2.1.2 Clinical project

The study is based on a clinical project aimed at developing effective integrated healthcare for children under 3 years old with RPs, autism, and other coexisting NDDs and medical conditions. The clinical project was conducted (from 2021 to 2023) within a low-resource area, where an extremely large proportion (approximately 80%) of families have an immigrant background. The clinical project was founded by the Västra Götaland region. The target group consisted of approximately 1,600 children, ranging from birth to 3 years old, who were followed up within the child health services. Three child health units were a part of the clinical project, with a total of 12 child health nurses. General practitioners were responsible for medical assessment within the regular health program, and psychologists could be consulted. Primary care staff from child health services, specialist healthcare providers, and habilitation professionals were included in the collaborative project. In addition, the preschool administration, represented by special educators in the district, was also included.

The clinical project included educational efforts to increase professionals’ knowledge about early neurodevelopmental symptoms, persisting RPs, symptom mapping, assessments and interventions (see Table 1), and new methods for collaboration to ensure coherent care (see Table 2).

**Table 1:** Increased knowledge for healthcare and preschool staff.

Educational initiatives in child health services Four workshops (2 h each) with a focus on
Early symptoms of autism and/or other neurodevelopmental symptoms. Persisting RPs (see Supplementary Material 1). Mapping at child health services and follow-up. Extended health visit at 18 months including a focus on RPs and early symptoms of autism (see Supplementary Material 2) Early advice to parents and start of interventions for difficulties in social communication. Principles from the ESDM
Education on naturalistic interventions Psychologists within child health services and specialist care underwent online introduction to ESDM. Introduction workshop—ESDM (3 h) by certified ESDM therapists and opportunities for supervision to team members in the specialist team during the project
Educational initiatives for all preschool staff in the area working with children aged 1–3 years old (approximately 200) Digital education with a focus on “social communication difficulties in children aged 1–3 years old and strategies to guide them in daily activities.” Supervision for preschool staff was held by a specialist education teacher.

RP, regulatory problems; ESDM, Early Start Denver Model.

**Table 2:** Novel methods to gain improved collaboration for integrated healthcare.

Digital consultation—following a structured digital routine. Lasting for 30 min once a month.
• Child health nurses, a child health psychologist, and a paediatrician from specialist care participated.
• Two to three children were presented by the child health nurses according to a specific structure. Based on the consultation, plans for further assessment and interventions were made.
Consultation with a speech language pathologist at the child health service
• Two or three occasions per semester at each child health unit; consultations were offered as an opportunity for initial counselling for the staff and family members when there were questions about language development.
Extended health visit to the child health nurse for all children at 18 months
• The visit included a follow-up regarding RPs (see Supplementary Material 1) and the development of social communication. A checklist for follow-up of RPs and a five-item observation of the child's joint attention ability with the instrument JA-OBS was used (see Supplementary Material 2).
Specialist team assessment at the child health service
• Child health nurse invited and prepared the family and the team for the visit, where the child health psychologist and the paediatrician from specialist care participated. The team met approximately 5–6 times/a year.
Early interventions in primary care
• After the child health team assessment, the child health psychologist offered a follow-up for the parents within 1 week. If there was a suspicion of autism, the child health psychologist had an additional 1–2 appointments with the family to guide the parents in strategies based on the ESDM before further investigation in specialist care.

RP, regulatory problem (see Supplementary Material 1 for the definition used); JA-OBS, joint attention observation; ESDM, Early Start Denver Model.

At monthly digital consultations for each child health unit (30 min), joint assessment and planning were possible for children with concerns (see Table 2). Novel methods that supported the joint work included templates for mapping RP symptoms and a template for an extended 18-month health visit to the child health nurse (Table 2; Supplementary Material 2). There were also opportunities, every second month, for assessments by a specialist child health team (consisting of the responsible child health nurse, a child health psychologist, and a paediatrician from specialist care). The initial interventions started via child health services, where the child health psychologist offered a follow-up for the family within 1 week, offering more information and support based on the individual needs of each child and family. In cases of suspected autism, an additional one to two appointments were offered to guide parents in strategies based on the ESDM before further investigation in specialist healthcare.

The multiprofessional evaluation was based on the referral from child health services, including medical records from the neonatal period. In the first meeting, a survey of the family's social situation, current needs, and the child's preschool situation was conducted. The evaluation included (i) an observation of the child during play activities and a parental interview during a team visit with the paediatrician and psychologist, (ii) a physical developmental examination of the child, followed by complementary medical investigations based on the paediatrician's assessment, (iii) psychological assessments using standardized diagnostic instruments, and (iv) information from preschool, obtained from the special education teacher, along with an observation of the child in the preschool.

The goal was to assess the child within 4–6 weeks, summarize the investigation and diagnostic assessment results with the parents, and create an intervention plan with them. The same team was responsible for treatment efforts and follow-up during the 1-year period within the local team. For children with autism, the interventions were based on the ESDM (22). The parents were coached in strategies for their child twice a month. Meetings with the preschool were held to share information and involve the preschool staff as much as possible in the goals. According to the child’s individual needs and coexisting conditions, other treatments and interventions were provided.

### 2.2 Data collection

Data, including waiting times for children referred from primary care for evaluations due to a suspicion of a neurodevelopmental disorder and the age at diagnosis, were collected retrospectively and prospectively from medical statistics in healthcare.

### 2.3 Participants’ qualitative part

Eight child health nurses out of 12 possible nurses working at the included CHS were recruited for the interviews. The inclusion criterion was that the nurse needed to have been part of the clinical project. In total, six interviews were conducted; two of these interviews included a pair of two nurses each.

### 2.4 Data collection qualitative part

The interviews were conducted by author EC, who was not a part of the specialist team. The interviews were semi-structured, audio-recorded, and subsequently transcribed verbatim (43). The interviews were conducted using a question guide developed by two of the authors (EC and GN). The questions were informed by prior clinical experiences and previous research. The guide included questions such as: “What does the care process look like for these children and their parents?, How do you perceive your opportunities to support the parents?”. The first interview served as a pilot to evaluate the questions and refine them if necessary. However, no changes were made.

### 2.5 Qualitative data analysis interviews with nurses

The author EC transcribed all interviews and then read the transcriptions multiple times to gain an overview before analyzing and interpreting the data using content analysis as described by Graneheim and Lundman (44). The structured analysis involved sorting the material into meaning units. After identifying all meaning units, the author labelled each unit with a code. The analysis involved ongoing discussions among the authors to ensure methodological rigour and the trustworthiness of the results. The authors reflected on and discussed similarities and differences among codes, which led to the development of categories and subcategories. They continually reviewed and refined interpretations of these categories (45).

### 2.6 Ethics

This study was approved by the Swedish Ethical Review Authority (Dnr 2022-02070-01). All participating nurses received oral and written information and provided written informed consent. To ensure anonymity, names were removed.

## 3 Results

The results are divided into two parts: the quantitative findings, which include data from medical statistical charts, and the qualitative findings, derived from interviews with healthcare nurses.

### 3.1 Healthcare statistics

Table 3 shows the time from detection of neurodevelopmental symptoms to diagnosis and the start of interventions, before and after the project started. Fifty-nine children (28% girls) were referred to the multiprofessional team with suspected autism and/or other neurodevelopmental disorders. Among them, 55 were diagnosed with autism and other coexisting conditions, while the other four were diagnosed with “non-autism” neurodevelopmental disorders. The average age at diagnosis was 27 months.

**Table 3:** Care chain—from detection to interventions.

	Age at referral from child health services M (SD)	Age at diagnosis in specialist care M (SD)	Start of intervention M (SD)
Before the project	38 months^a^	44 months^a^	18 months^a^ after diagnosis (approximately 60 months of age)
During the project	27 (5.2) months	31 (5.2) months	27 (5.2) months (at referral)

^a^ SD not available.

When persistent RPs were identified, mapping and assessments were conducted at the child health service by the nurse and the child health specialist team. Most families were offered support and follow-up within the child health services, while some were referred to the child health specialist team. A total of 13 children with RPs were referred to the specialist team for medical assessment and interventions towards more severe RPs, in most cases feeding/eating problems. The average age at referral or at the time of assessment in specialist care was 15 months.

### 3.2 Qualitative results

Findings from the qualitative data conducted through interviews showed three categories: (1) new and increased knowledge, (2) great importance for every child and family, and (3) efficient method with fewer gaps, which has further potential for development, with eight subcategories presented in Table 4.

**Table 4:** Categories and subcategories from the qualitative interviews with child health nurses.

Categories	Subcategories
1. New and increased knowledge	Confidence within the work environment
	Early detection and early interventions
	Good collaboration—a key to success
2. Great importance to every child and family	Crucial for all families—including those who do not raise specific concerns
	Easier to motivate parents—but still challenging
3. Efficient method with fewer gaps has further potential for development	Less resource-intensive in the long run
	Team is a strength—access to a wider range of professionals
	New approach ensures seamless transitions in healthcare and in collaboration with preschools

### 3.3 New and increased knowledge

The category “new and increased knowledge” captured the nurses’ experiences of gaining new knowledge through the project. This encompassed both the development and implementation of new methods and structures, as well as the educational part of the project, which included workshops and discussions.

#### 3.3.1 Confidence within the work environment

The nurses indicated that through collaboration and participation in the project, they felt an increased sense of confidence in their professional roles. The collaboration between primary care and specialist care made the child health nurses feel supported and enhanced their knowledge and confidence. Continuous consultation and team collaboration were critical improvements, allowing the included staff to use a “common” language.

“It makes it easier for me if I involve the parents, I also think it’s a kind of security for me.”

#### 3.3.2 Early detection and early interventions

The nurses shared their experiences of detecting difficulties earlier, allowing them to support and reach out to families sooner. They expressed having more effective tools for discussing their observations with parents. The increased knowledge about early signs of developmental difficulties in children made the nurses more confident about discussing their worries with parents and more likely to see early signs of developmental difficulties.

“That one can find them [early signs of developmental difficulties] … and talk about it in a better way with the parents.”

#### 3.3.3 Good collaboration—a key to success

All included healthcare and preschool staff work towards the same goal. Nurses expressed that the new routines and close contact with the team and project leader were crucial to increasing collaboration and minimizing gaps. Through collaborative work and educational sessions, the nurses increased their knowledge and confidence.

“That everyone participates with great interest and commitment … that it is a task that one finds exciting and motivating”.

### 3.4 Great importance to every child and family

The category “great importance to every child and family” consisted of two subcategories focusing on children and families. According to the nurses, these subcategories demonstrated that the new way of working was beneficial for all families, including those whose children were of concern and those whole children who developed as expected.

#### 3.4.1 Crucial for all families—including those who do not raise specific concerns

The increased knowledge gained through the project provided the nurses with “tools” in their “toolbox” for assessing children who raised concerns; these tools proved to be useful in their general work as well. The “new” structure and methods were helpful for all children, not just those of concern. Asking parents more about RPs proved beneficial for several families. Although the nurses mentioned that they had previously inquired about RPs, they now had a more structured approach to addressing these issues. In addition, they had resources to support families by offering digital consultations and/or referrals to specialist CHS.

“ I also think that with the knowledge that has increased within oneself, it also helps other families where there is no concern.”

#### 3.4.2 Easier to motivate parents—but still challenging

The new tools, shorter waiting times, routines for specialist assessments in close connection with nurse visits, and the possibility to offer psychological support for parents facilitated motivational work with parents. It helped them motivate parents to accept further referrals. However, they described that it could still be challenging to help parents understand the importance of sending a referral. There were different opinions regarding early detection between nurses; some thought it affected parents’ motivation, since some parents did not worry since there were several years until their 2-year-old started school.

“Those who have been uncertain and had to wait a bit and receive support in between, well … many say yes afterwards.”

### 3.5 Efficient method with fewer gaps has further potential for development

The category “efficient method with fewer gaps” highlights the potential for further development; it focused on how the new methods worked, the benefits of teamwork, how the new structures improved healthcare transitions, and making collaboration with preschools easier and more effective.

#### 3.5.1 Less resource-intensive in the long run

The improved healthcare chain with reduced waiting times and fewer gaps was more resource-effective due to the nurses’ experiences. Significant time and resource savings were achieved by preventing families from waiting and ensuring they received support and/or interventions as soon as concern about their child's development was identified. The nurses described that they had got more “tools” and knowledge of how to support families. *“*In that case, it would have required even more resources later on, so I think it's a resource-saving measure overall.”

#### 3.5.2 Team is a strength—access to a wider range of professionals

Having access to a team, i.e., the possibility to consult a doctor or psychologist, was beneficial and valuable for daily work, not least when being new at work. The nurses said that the fact that they worked together with other professions made them gain new knowledge and develop their professional skills.

“I feel like I have very good support from them. Especially if you haven’t been working for very long.”

#### 3.5.3 A new approach ensures easy and smooth transitions in healthcare and in collaboration with preschools

The new structure and collaboration within the project made transitions smoother between healthcare levels and facilitated the nurses’ work. One key point highlighted by the nurses was that knowing parents could access support without long waiting times made it easier for them to discuss their concerns with the parents. It was also beneficial when collaborating with preschools and preschool staff.

## 4 Discussion

The aim of the study was to evaluate novel methods within a clinical collaborative project to address gaps in healthcare for young children with autism and other NDDs. An additional aim was to explore the experiences of new methods and structures in CHS from the perspectives of child health nurses.

The results showed that the mean age for identifying autism signs and referring children from CHS to specialist care decreased by 11 months, from 38 to 27 months. The shortened wait time for specialist care evaluations resulted in a mean age of autism diagnosis of 31 months, compared to 44 months previously. Interventions were started already at the point of autism suspicion in primary care (27 months). The results showed a significant improvement regarding age for autism diagnosis and opportunities for interventions compared to the local average (60 months) for interventions. The results for age at diagnosis are also well below what globally is reported (18). The mean age for diagnosis is also significantly lower than global average (18). Several studies have highlighted the increased prevalence of autism in children from immigrant populations (38, 39, 40) and have also noted the risk of delayed diagnosis and lack of services for these children (46). As the current study was conducted in an immigrant population in a low-resource city district, the results that show access to healthcare with reduced waiting times are promising findings.

Research emphasizes the importance of early identification of autism, persistent RPs, and other early NDDs. Coexisting neurodevelopmental conditions are more the rule than the exception (30) and must be broadly assessed for targeted interventions. Specialization in care and organizational shortcomings, including long waiting lists, can, when symptoms have been identified, become an obstacle for assessments and targeted interventions for different simultaneous neurodevelopmental and medical conditions (29, 31). It may take several years after symptoms have been identified before targeted interventions can begin, which can entail significant consequences for the individual child and family. Novel methods, such as mapping templates for RPs and a new extended form for an 18-month developmental check-up, including the observation of joint attention ability, the joint attention observation (JA-OBS) (13), provided support in early identification of autism signs and/or other neurodevelopmental symptoms; these tools also facilitated collaboration with, and referrals to, specialist care. When persistent RPs were identified within CHS, most families were offered early interventions and follow-up within CHS, and some children were referred to specialist care for further medical assessments and treatment. The reason for referral to specialist care was, in most cases, feeding/eating problems, with the average mean age for referral being 15 months. The nurses reported the increased knowledge and structural approach to addressing early symptoms, including RPs, opportunities for team collaboration, and consultations with specialist care, as critical improvements.

The qualitative results showed three main categories. The first, *new and increased knowledge*, included descriptions from the nurses that knowledge of and the ability to identify early neurodevelopmental symptoms and medical conditions form the basis for timely assessments and interventions. The nurses described that the increased knowledge gained through initial educational investment and an ongoing opportunity for learning through new ways of collaborating with healthcare specialists played a decisive role. *increased knowledge and “common language”* were the basis for more effective cooperation in care to identify difficulties in children's development, engage in conversations with parents, and offer investigations and interventions. When collaborating with preschools and preschool staff, it was also beneficial to gain a “common” language.

The second main category, *great importance to every child and family*, addresses issues of and includes descriptions from the nurses that the new methods and structures were beneficial for children suspected of having NDDs, but they highlight its value for all children and families that took part in CHS. The first meetings within child health services could create conditions for building trust and forming an alliance with parents (47), which was very important when the child was referred for further investigation and interventions in specialist care. Earlier studies have highlighted the importance of assessing the individual needs of the child and family in a holistic way (30, 48, 49). The nurses described that the family and child's situation in preschools must be considered. In all interventions for the child, the alliance with the parents is crucial (47, 49).

The third main category, *Efficient method—fewer gaps—further potential for development*, the new structure and collaboration within the project made transitions smoother between different healthcare levels and facilitated the nurses’ work. One key point highlighted by the nurses was that knowing parents could access support, without long waiting times, made it easier for them to discuss their concerns with the parents. This finding is important for future guidance on improving and developing healthcare pathways for children and their families, which is in line with earlier-presented suggestions by Mendez et al. (4).

### 4.1 Strengths and limitations

To strengthen the trustworthiness, an interview guide was employed to ensure that the questions were aligned with the study's aim, strengthening dependability. Confirmability was ensured by using quotes from the interviews to illustrate subcategories, and reflexivity was maintained throughout continuous discussions among the authors during analysis (50).

This study did not include the parents’ experiences, nor did it explore the children's developmental trajectories or their future access to interventions. For replication and extension beyond the current population, a larger study would be necessary to learn more about its possibility for generalizations in other settings.

### 4.2 Future research

In a future study, the prevalence of RPs, their course, and any underlying and coexisting conditions will be examined. Moreover, while health-economic benefits were not within the scope of this study, they represent an important and interesting avenue for further exploration. The clinical experiences within specialist care suggested that most families were well prepared when they came with their children for the investigations. Many parents expressed that they had already received much help from the child health team and learned strategies to help their child. This would be interesting for a future study.

### 4.3 Conclusion

Professionals’ increased knowledge of early symptoms and neurodevelopmental concerns in preschool children, coupled with well-structured healthcare that foster close collaboration, made it possible to bridge the gaps and provide young children and their families with early assessments and essential interventions. The results of our study point towards better opportunities for integrated healthcare and collaboration with families and preschools.

## Data Availability

The raw data supporting the conclusions of this article will be made available by the authors without undue reservation.
