# Supplementary material for: Bridging gaps in healthcare: child health services and specialist care collaboration for young children with autism and coexisting conditions
Source: Front Pediatr. 2025 Feb 6;13:1501650. doi: 10.3389/fped.2025.1501650 (PMC11839616; doi:10.3389/fped.2025.1501650)
Supplement: Supplementary file 1 [file Datasheet1.docx]

# Supplementary material 1.

# **Definition of persistent substantial regulatory problems (RP), in children after 3 months of age**

Applies to children 3- 18 months of age

Regulatory problems (during more than 2 weeks after 3 months of age) and causes special concern at child-health visit for the family and/or, and leads to support/intervention and surveillance through child-health services.

RP crying = burst> 2 episodes/day and/or the child cries > 1 hour/day and shows discomfort> 50 %/day

RP sleeping= it takes >1 hour to sleep and/or the child cannot sleep more than 3 hours in a row.

RP feeding =the feeding situation is difficult regarding something of these examples:

The child does not show interest in eating, need to be forced to get enough energy/food intake.
After 6 months still restricted and selective or other concern regarding the child’s food intake where support through child-health or specialist-care is indicated.

The definitions are based on definitions used in international studies of ”Persisting Regulatory Problems” (Hemmi et al., 2011, Cook et al., 2019, Olsen et al., 2019)

RP= Regulatory problems.

Supplementary material 2.

**Health-visit within child-health services at 18 months- Små barn Nordost**

Ordinary 18 months health-visit is documented as usual.

Complementary assessment regarding Regulatory problems (RP) and Joint-Attention Observation (JA-OBS)

***Shown RP* at previous child-health visit?***

YES

NO

**Specialist child-health team assessment is planned**

- If persistent RP or new debuted RP
- If screened positive with JA- OBS and/or other concern regarding the child’s psychomotor development

*RP=regulatory problems,

**Joint Attention (JA)-OBS at 18 months (if yes-on the question check the box)**

**□** Does the child respond to their name?

**□** Does the child seek eye-contact with you?

**□** Can the child follow when you point at something farther away in the room?

**□** Does the child use their own index finger to point at something?

**□** Can the child engage to simple pretend game?

If check in 2 or more boxes in JA-OBS complement with MCHAT-R and inform parents about the possibility of assessment at the specialist – child health team

- IF parents’ consent are given information from preschool is collected

No previous known RP, but RP was shown at 18 months. Mapping is performed:

Sleep □

Feeding □

Crying □

RP persisting:

What RP?

Sleep □

Feeding □

Crying □

RP ceased:

What RP?

Sleep □

Feeding □

Crying □
